# Managing unwarranted variation in hospital care – findings from a regional audit in Norway

**DOI:** 10.1007/s43999-023-00033-7

**Published:** 2023-11-15

**Authors:** H. P. Eide, P. Barach, E. Søreide, C. Thoresen, O. Tjomsland

**Affiliations:** 1 Southern and Eastern Norway Regional Health Authority, Parkgata 36, Hamar, 2317 Norway; 2 Department of Pediatrics, Wayne State University, Detroit, MI USA; 3 Jefferson College of Population Health, Philadelphia, PA USA; 4 Sigmund Freud University, Vienna, Austria; 5 University of Stavanger, Stavanger, Norway; 6 Stavanger University Hospital, Stavanger, Norway

**Keywords:** Quality improvement, Quality of health care, Governing board, Leadership, Outcome assessment, Health care

## Abstract

**Background/Aim:**

There has been increasing focus and research over the past decades on defining, identifying, visualizing and reducing unwarranted clinical variation in clinical practice. Both clinician-driven initiatives such as the US based “Choosing Wisely” campaign and the top-down driven “Evidence-based intervention programme (EBI)” launched by NHS UK to improve quality of care by reducing unnecessary interventions have shown marginal results. We present the findings from a mixed-methods audit performed to evaluate the compliance by senior hospital leaders of a new regional strategy to reduce unwarranted variation in outcomes and utilization rates.

**Methods:**

Seventy-five mid- to senior-division and department leaders from eight hospital trusts in South-Eastern Norway Regional Trust (HSO) were invited to participate in evaluating the response and compliance of the regional leadership strategy for reducing unwarranted variation in patient outcomes and service utilization rates.

**Results:**

The audit revealed that the aim of reducing unwanted variation was not clearly communicated by senior HSO management. There was varying use of data from the national quality registers and health atlases for quality improvement. One third of the clinical leaders reported a lack of scrutiny of their work and were insufficiently aware of the HSO’s top-management and the hospital’s Boards strategic expectations about the importance of reducing unwarranted variation in their hospital utilization.

**Conclusions:**

We found that the strategic aim of reducing unwanted clinical variation was not clearly communicated by senior HSO management to hospital boards and senior management.

The hospitals could benefit from a better understanding of causes of variation by strengthening their efforts to reduce unwarranted variation in utilization rates as a key element in improving health care quality and patient safety. The findings of the audit are relevant for other healthcare organizations trying to improve their quality and reduce unnecessary variation.

## Introduction

Healthcare systems face challenges in tackling variations in patient care and outcomes [1]. Several initiatives have been launched to monitor and reduce unwarranted variation in utilization rates and outcomes of medical and surgical interventions. Both clinician-driven initiatives such as the USA clinician driven “Choosing Wisely” campaign and the more “top-down” evidence-based intervention programme (EBI) launched by NHS England to improve quality of care by reducing unnecessary interventions have shown mixed results [2, 3].

Utilization rates for medical and surgical interventions in Norway vary between health regions and hospitals, which is well documented in the national Health Atlas comparisons [4]. Our previous published experience demonstrates how indicators and utilization rates can help identify and implement measures to reduce unwarranted variations in mortality and utilization rates [5, 6]. The 2018 strategic document of South-Eastern Norway Regional Trust (HSO) named “Regional Development Plan 2035” defined the reduction of unwarranted variation as one of five prioritized objectives [7]. HSO uses an internal audit group reporting directly to the Board with an independent and objective confirmation- and advisory function responsible for internal audits in the hospitals owned by the Norwegian Southeastern regional health authority.

There are several known tools to reduce unwarranted variation, such as guideline-improvement, shared decision-making and clinical audits [1]. Clinical audit and feedback entail reviewing clinical performance against explicit standards and delivering feedback to enable data driven improvement. Several national audits have been successful in driving improvement and reducing variations in care, such as for stroke and lung cancer, but progress has also been slower than hoped for in other aspects of care [8, 9].

The objective of the audits was selected based on inputs from the hospital Boards in the region and carried out in accordance with international standards for the professional practice of internal auditing set by The Institute of Internal Auditors (IIA) [10]. Several hospital Boards suggested in 2021 to audit the compliance of the regional strategy for reducing unwarranted variation in outcome and utilization rates [7]. The objectives of the internal audit were to investigate which data sources and targets managers and clinical leaders use to monitor and reduce unwarranted variation of utilization rates and outcomes, and whether managers perceived that reducing unwarranted variation was central to top management’s performance standards and aligned with the hospital Board’s clinical governance framework. The aim of this paper is to present the main findings of the audit in driving improvement and reducing unwarranted variations in care quality.

## Methods

### Overview of the Norwegian healthcare system

The specialist health care system in Norway is predominantly a publicly funded universal health coverage system designed to care for about 5.5 million citizens and is provided by four regional health authorities, each responsible for delivering specialist healthcare in their regions. The Southeastern Region HSO provides specialist healthcare for approximately 3.1 million inhabitants representing 57% of the population in Norway. HSO is divided into 6 hospital catchment areas with a population of 150,000-500,000 each. The local health trusts within the region (seven trusts and two private non-for-profit hospitals) are legal entities governed by independent boards with an overall responsibility for the clinical services they provide.

### Study participants

Eighty-two mid- to senior-division and department leaders (level 2 and 3) from the participant hospital trusts were invited to complete a validated questionnaire regarding overseeing care for patients with the following diagnoses and procedures:

- diabetes
- cerebral stroke
- myocardial infarction
- hip fracture
- joint prosthesis
- patients undergoing day care surgery

### Study measures

#### Quantitative analysis

An audit was developed in accordance with the international standards for the professional practice of internal auditing by The Institute of Internal Auditors (IIA) [10], The audit included:

I) A cross-sectional survey of HSO mid-, and senior-level managers; and.
II) Interviews of Hospital CMO’s and other senior leaders.

The questionnaires consisted of two parts:

1. The first part included demographic questions, with an online questionnaire followed by an electronic mail (e-mail). The topics that guided the questionnaire development included:

- Is reducing unwarranted variation in utilization rates of medical interventions communicated to the clinical leaders as part of clinical governance from the board and hospital top management?
- Are targets for reducing unwarranted variation defined and communicated to clinical leaders?
- Are the defined targets perceived as balanced and realistic?
- Are the set targets for activity vs. quality and utilization rates conflicting?
- Do you have the right means for executing this responsibility?
- Which data sources and targets do you use to monitor quality and utilization rates?
  - ◦ National health atlases [4].
  - ◦ Data from national Quality Registries [11].
- In which areas are hospital division leaders following up on subordinate departments, and how often is this done?
- In which areas does the managing director follow up on clinic/division managers, and how often is this done?

#### Qualitative analysis

 The second part consisted of semi-structured individual key stakeholder qualitative interviews with three Chief Medical Officers (CMOs) and eight division leaders using pretested piloted interview guides. Most interviews were done in person; a few were conducted remotely, digitally due to COVID-19 social distancing restrictions. All interviews were documented and transcribed verbatim in Norwegian in a standardized format. The interviewer was trained in qualitative research methods and supervised by the study’s research staff. No relationship was established between the interviewer and the study’s participants before the study commencement. The research team discussed the emerging key themes during the interview, and data richness and some of the interviews were analyzed in parallel with the data collection. We derived new hypotheses as a result of this ongoing data analysis.

### Procedure

The survey was conducted in eight of the HSO’s public health trusts and in two private non-for-profit hospitals with full time Norwegian managers and CMO’s between February and September 2022. The survey was closed in September 2022.

### Data analysis

All the interviews were transcribed and analyzed using a thematic analysis method based on grounded theory. The analysis included incorporating deductive themes arising from research topics and based on an exhaustive literature review of clinical audit and clinic variation together with inductive theme that emerged from the data.

### Ethics

As the audit was a part of the internal audit-program of HSO, approval from the Regional Ethical Committee was not needed.

## Results

**Fig. 1:**
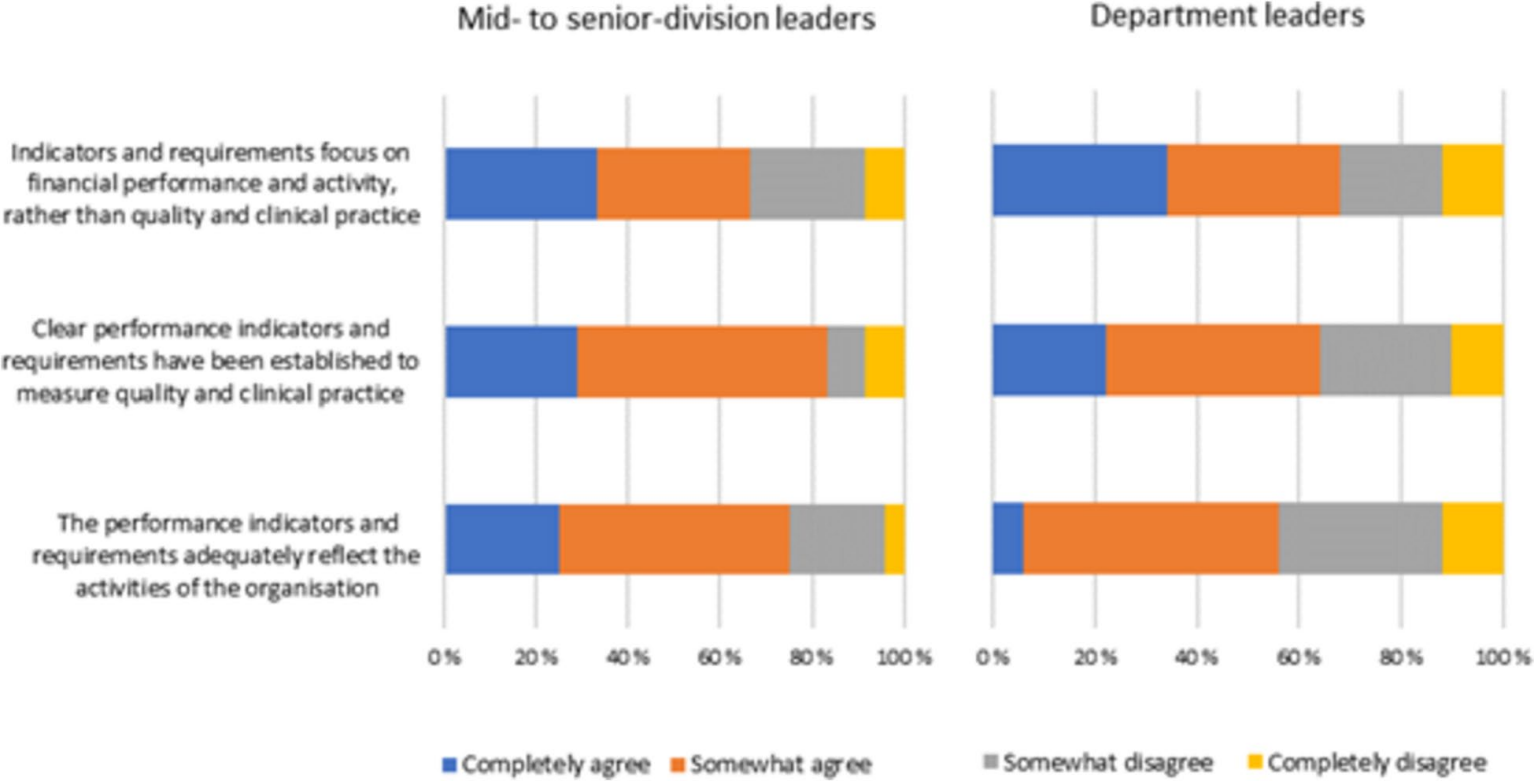
The performance indicators and requirements adequately reflect the activities of the organization

**Fig. 2:**
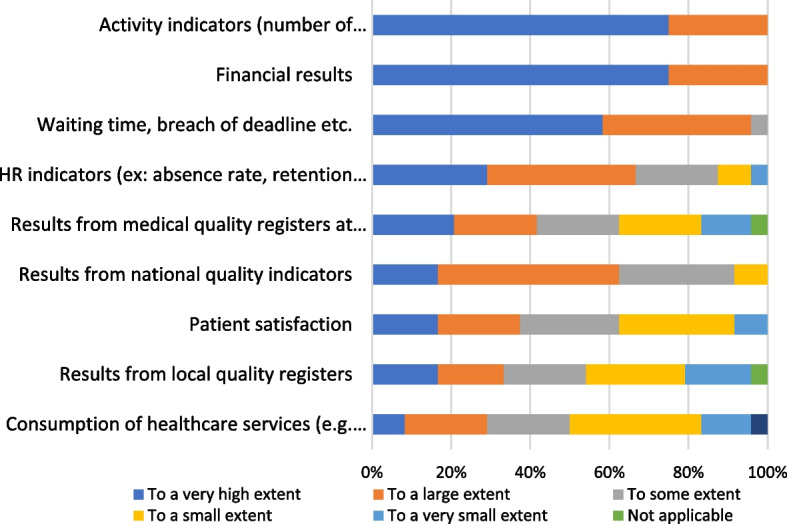
Clear performance indicators and requirements have been established to measure quality and clinical practice

**Fig. 3:**
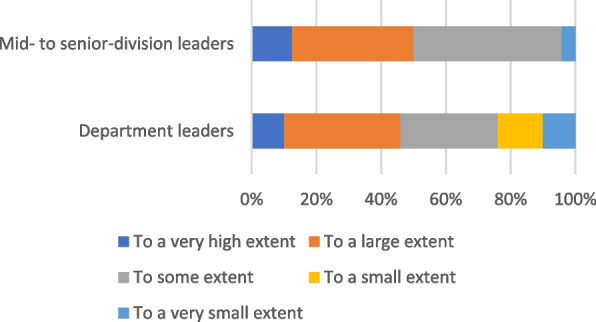
Perception of the relevance of activity indicators (number of consultations, inpatient days, etc.)

Seventy-five of the 82 invited managers (91%) responded to the questionnaire. Several themes emerged from the interviews and the questionnaire analysis. The themes included:

- Many hospitals had established structures and processes to achieve overall goals and requirements, including reducing unwarranted variation in utilization rates and outcomes. On a scale from 1 to 5, the included hospitals were assessed on a maturity scale to be evenly distributed between 2 and 4. Some of the hospitals had not progressed as far as expected in this work, which could have increased the risks for patients receiving treatment not according with best practice.
- Reducing unwanted variation was not always clearly communicated by senior management, 1/3 of the managers responded that the goal of reducing unwanted variation had not been clearly communicated by the top management.
- There was varying uses of data from the national quality registers and health atlases for quality improvement, 1/3 of the managers reported that they did not use available information from national quality registers and health atlases in their efforts to ensure good quality and services in patient treatment.
- One third of the clinical leaders experienced lack of scrutiny of their work and were insufficiently aware of HSO’s top-management and hospital boards strategic expectations regarding the importance of reducing unwarranted hospital utilization variation in care quality in daily medical practice.
- The majority of department leaders as well as the mid to senior department leaders thought that the performance indicators and requirements adequately reflect the activities of the organization (completely-/somehow agree) (Fig. 1).

- There were mixed perceptions regarding whether performance indicators and requirements had been established to measure quality and clinical practice (Fig. 2).

- There were various perceptions on the relevance of the activity indicators (number of consultations, inpatient days, etc.) (Fig. 3).

The audit revealed a positive trend in terms of number of local health trusts including quality of care and utilization management as a subject of close scrutiny of all management levels and the development of improvement strategies.

## Discussion

The audit revealed that reducing unwanted variation of clinical practice was not always clearly communicated by senior management to managers and clinicians despite the fact that the majority of the hospital trusts had structures and processes in place to reduce unwarranted utilization rates. Many clinical leaders experienced little scrutiny or oversight of their work to address and reduce causes of variation and were insufficiently aware of the HSO’s senior management and hospital Board’s strategic expectations on the importance of reducing unwarranted hospital utilization variation.

It was recommended that the hospitals included in the audit should strengthen their efforts to reduce their unwarranted variation in utilization rates. This should be central to their managing quality and patient safety and that hospital senior management and Boards should focus on reducing unwarranted variation in hospital outcomes and utilization rates of medical interventions.

### Limitations

Our study has several limitations. First, the study results reflect the Norwegian health system, which may not be generalized to other countries with their distinct health delivery systems, comprising unique legislative and organizational characteristics, and within different clinical and political settings. Second, although the themes seemed consistent across the interviewees, and the influence of the varying systems on the findings was frequently discussed during the data collection and analysis, the local and specific impacts of these cultural barriers may have been under-appreciated. Third, the interviews were transcribed from Norwegian, the native language in Norway. This may have increased the chances for variations in the interpretation of our data. We made all efforts to ensure methodological rigor and validity of the translations from Norwegian to English by using a standardized codebook, meeting frequently, sharing and comparing our results, and performing a pilot analysis.

### Recommendations and implications for practice and health policy

We believe that there is a need for hospital managers to better understand the causes of variation and that the findings of this audit may be relevant for other healthcare organizations seeking to improve the quality of medical care and reduce unwarranted interventions. Furthermore, we believe that the abundance of data presented to hospital Boards and senior management might contribute to a loss of focus on the targets of unwarranted variation in quality and patient safety. We think that presenting a limited selection of data reflecting clinical outcomes and utilization rates focusing on a deeper understanding of the variation causes can contribute to an increased focus and level of competence in members of hospital Boards and management to better address their governance and fiduciary duties.
